# Leptin Affects Life History Decisions in a Passerine Bird: A Field Experiment

**DOI:** 10.1371/journal.pone.0004602

**Published:** 2009-02-26

**Authors:** Mare Lõhmus, Mats Björklund

**Affiliations:** Department of Animal Ecology, Evolutionary Biology Centre, Uppsala University, Uppsala, Sweden; Lund University, Sweden

## Abstract

**Background:**

Organisms face trade-offs regarding their life-history strategies, such as decisions of single or multiple broods within a year. In passerines displaying facultative multiple breeding, the probability of laying a second clutch is influenced by several life-history factors. However, information about the mechanistic background of these trade-offs is largely lacking. Leptin is a protein hormone produced by white fat cells, and acts as a signal between peripheral energy depots and the central nervous system. In addition, leptin affects cells at all levels of the reproductive axis and plays a critical role in regulating the allocation of metabolic energy to reproduction. As such, it is possible that leptin levels influence the decision of whether or not to invest time and energy into a second clutch. Accordingly, we expect a treatment with exogenous leptin to result in an increased number of second broods.

**Methodology/Principal Findings:**

At a later stage during the first brood, female great tits were treated either with long-term leptin-filled cholesterol pellets (the experimental birds) or with pellets containing only cholesterol (the control birds). We found that leptin-treated females were significantly more likely to have a second brood and that the earlier females were more likely to lay a second clutch than the late females.

**Conclusions/Significance:**

As both timing of first brood and treatment with leptin were important in the decision of having multiple broods, the trade-offs involved in the breeding strategy most likely depend on multiple factors. Presumably leptin has evolved as a signal of energy supply status to regulate the release of reproductive hormones so that reproduction is coordinated with periods of sufficient nutrients. This study investigated the role of leptin as a mediator between energy resources and reproductive output, providing a fundamentally new insight into how trade-offs work on a functional basis.

## Introduction

Life histories of organisms can be considered as strategies that optimize reproductive effort and allocation of resources for reproduction [1]. Any constraints in resource allocation processes generate trade-offs between an individual's survival and its investment in offspring [1], [2]. Even if reproductive effort is not the only aspect of life history, other factors, such as survival, only contribute to fitness if the prolonged survival results in increased reproduction.

Passerines of northern latitudes generally display a strong negative relationship between timing of the breeding season and reproductive output, either because of changes in the seasonal food supply and/or a lower quality and survival of the later-hatched chicks [3]–[9]. Similarly, in bird species with facultative multiple breeding, the first and the second breeding attempts often differ in clutch size, egg size and fledging success [3], [10]–[14]. Consequently, there must be several trade-offs related to the allocation of reproductive investment in multiple breeding attempts [11]. Studies investigating these trade-offs within geographic locations have shown that the probability of laying a second brood in great tits can be influenced by the type of habitat, the age of the female, population density and time of laying the first clutch [3], [10]–[14].

The endocrine system is important for mediating the allocation of energy to breeding effort at the proximate level. A myriad of hormones are directly or indirectly involved in the regulation of reproduction; however, the hormone leptin seems to be the most direct link between fat/metabolism and reproduction [15]. Leptin is a protein hormone that is primarily produced by white fat cells and circulates in the plasma at levels that correlate with body fat content. Besides being an important regulator of food intake and metabolism, leptin also affects cells at all levels of the reproductive axis [15], [16]. From mammalian experiments it is known that leptin stimulates the secretion of gonadotropin releasing hormone from the hypothalamus and the release of follicle stimulating hormone and luteinizing hormone from the anterior pituitary. Leptin has also been found to exert endocrine and/or direct paracrine effects on the gonadal organs thereby influencing follicle maturation and spermatogenesis [15], [17], [18]. As a threshold level of fat is vital for normal puberty and fertility in several mammalian species [15], [16], [18], leptin, as a messenger between peripheral energy depots and central nervous system, may play a critical role in regulating the allocation of metabolic energy to reproduction.

The great tit (*Parus major*) is a common breeding passerine in Europe [19]. Despite apparent ecological similarity between populations from different locations, however, there are remarkable differences in breeding strategies. For example, great tits nesting on Gotland (Sweden) have a low incidence of second broods (20% [14]; about 5% in 2007, pers. obs.), whereas populations of the same species at nearly the same latitude in south-western Estonia more frequently lay a second brood (around 50% [11]; about 75% in 2007, R. Mänd pers. comm.).

Since the level of energy resources is an important factor in functional reproductive endocrinology [15] it is likely that the difference in occurrence of second broods in great tit is influenced by local food supply and energy reserves of individuals. Furthermore, since the amount of body fat correlates with the levels of circulating leptin, higher secretion of leptin may increase the possibility that an individual will lay a second brood. Consequently, we can hypothesize that leptin supplementation may result in an elevated probability of second clutches.

When testing the influence of energy resources on breeding effort, researchers most often manipulate the food supply by supplementary feeding. This method, however, has been shown to have some side effects [9]. An alternative to testing the reduced energy availability hypothesis would be to manipulate the females' perception of their energy status without changing food availability. Therefore, to test whether treatment with leptin may result in an elevated probability of second clutches we inserted long-term pellets containing either recombinant chicken leptin or placebo as a control subcutaneously into female great tits at the end of their first breeding. If leptin is an important proximate cue for reproductive decisions, we expect to find a higher frequency of second broods in treated females compared to the control group. In this way, we directly manipulate the females' perception of her energy status without changing food availability.

## Results

**Figure 1:**
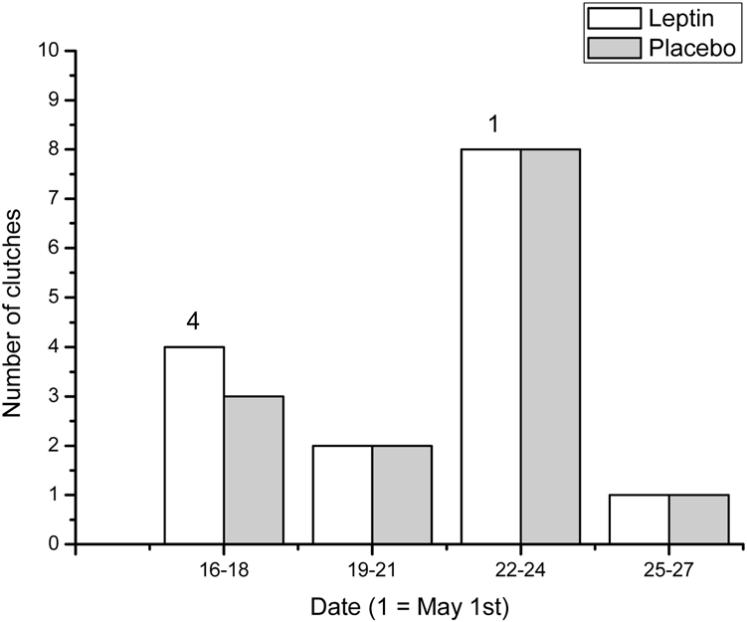
Hatching dates (in three-day bins) of leptin-treated and placebo-treated females. Numbers represent females that laid second broods.

Treatment with leptin had a significant effect on reproductive decision, with five (33%) leptin-treated and no (0%) control-treated females (P = 0.025, Fisher's Exact test) starting a second clutch approximately 5 to 7 days after the treatment. There was also a significant effect of date of the first laid egg of the first clutch on the probability of laying a second clutch (Fig 1). Earlier females were more likely to lay a second brood; the median date of the first egg was 27^th^ of April for females that laid a second clutch, and 4^th^ of May for females that did not (Mann-Whitney U = 11.5, P = 0.0051). There was also a significant difference in date of hatching between the females that started a second clutch and those that did not (Mann-Whitney U = 21.5, P = 0.023). When we restricted the analysis to leptin-treated females only, the effect of timing was the same (Mann-Whitney U = 4.5, P = 0.012). Both treatment and timing were important independently (Treatment χ^2^ = 7.90, df = 1, P = 0.0017, Date χ^2^ = 7.15, df = 1, P = 0.0075, logistic regression using hatching date as in Fig 1). Number of offspring was not significant when treatment and date of hatching was included in the logistic regression (χ^2^ = 3.27, df = 1, P = 0.070). Females that started a second clutch did not differ from females that did not start a second clutch, in terms of tarsus length (F_1,26_ = 0.79, P = 0.38), wing length (F_1,26_ = 0.053, P = 0.82) or mass (F_1,27_ = 0.40, P = 0.53). One of the five females that started a second clutch was a first-year female, whereas the other four females were older. Females that started a second clutch had a median of six offspring in their first clutch, whereas females that did not start a second clutch had a median of seven offspring (Mann-Whitney U = 30.5, P = 0.088; when only leptin-treated females were included the median values were the same, but P = 0.098).

## Discussion

Our main result was in accordance with the prediction that leptin implantation increases the probability of a second brood. This decision, however, seemed to be affected by a time component as the birds that laid a second clutch had started their first one significantly earlier than the ones that did not lay a second clutch. Consequently, there must be several trade-offs related to the allocation of reproductive investment in multiple breeding attempts [3], [4], [11], 12.

Our results suggested that we successfully manipulated the birds to ‘believe’ that they had enough resources to start a second brood, and in that way pushed them to lay a second clutch. In late breeders, the manipulation may have increased the perception of energy storage, but not enough to cross the threshold. Studies investigating these trade-offs have shown that the probability of laying a second brood in tits can be influenced by the type of habitat, the age of the female, size of the first clutch, population density and by the time of laying the first clutch [3], [11]–[14], [20], [21]. To our knowledge, our study is the first to show a possible adaptive effect of leptin as a mechanistic cue in a reproductive trade-off. It is obvious, however, that the trade-offs are complex since timing was also found to be an important factor in the decision of multiple broods.

The effect of exogenous leptin on the decision of laying a second brood may work on two levels: first, by mediating the neuro-endocrine signaling and making the birds “believe” that they were fatter than they actually were, and second, by the general stimulating effect of leptin on reproductive hormone release. It is nearly impossible to separate between these effects of leptin in present study. However, most researchers in the area seem to share the view that after leptin levels have reached a certain threshold value (equal to a certain amount of stored fat) an additional increase in this hormone does not involve any further endocrine advantages [15]. Since there was no difference in weight between the two treatment groups (meaning that both groups were equally “ready”), it may imply that the nutritional information mediator effect of leptin was more important in this study than its effect on the release of reproductive hormones. As leptin levels also affect other aspects of the physiology, such as the immune system [22], multiple effects of leptin treatment may influence the decision of laying a second brood.

Our results provide support for the hypothesis that a low incidence of second broods is at least partly due to resource availability. It is also possible that the female great tits on Gotland are not adapted to lay a second clutch, and therefore do not store sufficient fat even if the environment provided enough nutritional resources.

Other studies have shown that the size of the first brood is an important factor determining the probability of a second brood [14], [20], [21]. In our study, clutch size was not significantly (but only marginally) different between those that did or did not start a second clutch. Given the low sample size, the power of our test in this case is low. The leptin-treated female that started a second clutch very late (see Fig 1) had a first clutch of only four offspring. Although this is only circumstantial evidence, it does suggests that the investment in the first clutch may be important in the decision to start a second clutch, as previously shown multiple times in this species [14], [20], [21].

Many species face seasonal variation in the food supply, and breeding generally occurs during the peak of prey availability. However, although several new studies question the importance of available energy supply as a limiting factor in egg laying [23], the availability of food (usually caterpillars) required for feeding chicks has still been hypothesized to have some effect upon the formation of breeding strategies [24]. It has also been observed that parental weight loss during the first brood influences the likelihood of second broods in both great and blue tits [12]. Therefore, the probability of laying a second brood may be influenced by the amount of dispensable energy reserves after the first brood. Even though our study clearly supports that view, it is also obvious that the question is more complicated since although resource availability is important, it must be viewed in conjunction with other cues such as timing of breeding.

Within an evolutionary context, leptin may have evolved to function as a signal of energy supply to the hypothalamic-pituitary axis to regulate the release of reproductive hormones [17], since it is critical for organisms to coordinate reproduction with periods of nutrient sufficiency. It has been established that bird species have started to breed earlier as a result of the advanced onset of their major food sources (insects) due to increased spring temperatures. This results in a mismatch between maximum demand of food for the offspring and maximum food availability, leading to declining populations [25]–[28]. The rapid shift in breeding time can be understood at the mechanistic level if the role of leptin as a signal of immediate energy supply is seen as an adaptation to inform the individual of its reproductive potential. Since birds can only react to current food situations, changes in future resource availability cannot be handled at the proximate level. This in turn can easily result in the observed mismatch. In other words, what has evolved as a good adaptation under a given set of environmental conditions becomes a constraint when these conditions change quickly.

Leptin has rarely been investigated in terms of its evolutionary adaptiveness in non-production or non-domesticated organisms. This study investigated the role of leptin as a mediator between energy resources and reproductive output, providing a fundamentally new insight into how trade-offs work on a functional basis.

## Materials and Methods

The experiment was carried out in a well-established nest-box area on Gotland, in southern Sweden, in the spring of 2008. Nestboxes were checked regularly to obtain data on date of first egg, hatching time and breeding success throughout the breeding season. At the end of the first breeding period when chicks were 10 days old, small (ca 1×3 mm) control or leptin-treatment pellets were inserted subcutaneously into 14 control (P) and 15 experimental (L) females respectively. The leptin-treatment pellets released approximately 2 µg of recombinant chicken leptin/1 g body mass/day for 14 days. Recombinant chicken leptin had been previously purchased from Protein Laboratories Rehovot Ltd. Pellets used for experimental (cholesterol pellet containing leptin) or control (cholesterol only) treatment were produced by Innovative Research of America (Sarrasota, Florida, USA).

To avoid possible effects of lack of breeding sites (nestboxes), one nestbox next to the box where the females were breeding was sealed to prevent other species (e.g. flycatchers) to nest in these boxes. The seal was removed at the later stage of the first breeding, when most flycatchers were already settled. Overall, about 30% of all nestboxes in the area were unoccupied throughout the whole breeding period.

Insertion of the pellets was performed during the dark period of the day. Great tit females where caught from nestboxes and a small patch on their right breast muscle disinfected with ethanol. Pellets were inserted with tweezers through a small cut (ca 3 mm) in the skin covering the breast muscle. Additionally, all females were banded individually, measured and aged. Nests were matched in relation to the hatching date of the first clutch.

The treatment and control groups did not differ in terms of age (L: four first-year females and eleven older females; P: three first-year females, and eleven older females; X^2^ = 0.19, df = 1, P = 0.66), number of chicks (L: x (±SE) = 6.9 (0.43); P: x (±SE) = 7.1 (0.30), Mann-Whitney U = 100.5, P = 0.84), time of first egg (L: median = 12; P; median = 13; Mann-Whitney U = 88.5, P = 0.47, day 1 = April 20; time of hatching L: median = 52, P: median 53, Mann-Whitney U = 91.5, P = 0.56), tarsus length (L: x (±SE) = 22.26 (0.13); P: x (±SE) = 22.11 (0.15), F_1,26_ = 0.52, P = 0.48), wing length (L: x (±SE) = 72.7 (0.56); P: x (±SE) = 74.2 (0.63); F_1,26_ = 2.89, P = 0.10), or mass (L: x (±SE) = 18.2 (0.22); P: x (±SE) = 18.2 (0.18), F_1,27_ = 0.04, P = 0.83). We tested for homogeneity of variances in tarsus length, wing length and mass (Levene's test, P = 0.77, 0.21, and 0.77, respectively). Within-cell residuals did not deviate from normality (visual inspection of p-p plots). After the experiment the areas were checked carefully for the presence of second clutches.

## References

[1] Roff DA (2002). Life history evolution.

[2] Ellison PT (2003). Energetics and reproductive effort.. American Journal of Human Biology.

[3] Verboven N, Tinbergen JM, Verhulst S (2001). Food, reproductive success and multiple breeding in the Great Tit *Parus major*.. Ardea.

[4] Hansson B, Bensch S, Hasselquist D (2000). The quality and the timing hypotheses evaluated using data on great reed warblers.. Oikos.

[5] Wiktander U, Olsson O, Nilsson SG (2001). Annual and seasonal reproductive trends in the Lesser Spotted Woodpecker Dendrocopos minor.. Ibis.

[6] Drent RH, Daan S (1980). The prudent parent: energetic adjustments in avian breeding.. Ardea.

[7] Rowe L, Ludwig D, Schluter D (1994). Time, condition, and the seasonal decline of avian clutch size.. American Naturalist.

[8] Svensson E (1995). Avian reproductive timing: when should parents be prudent?. Animal Behavior.

[9] Verhulst S, Nilsson J-Å (2008). The timing of birds' breeding seasons: a review of experiments that manipulated timing of breeding.. Philosophical Transactions of the Royal Society B.

[10] Brinkhof MWG, Cave AJ, Daan S, Perdeck AC (2002). Timing of current reproduction directly affects future reproductive output in European coots.. Evolution.

[11] Mägi M, Mänd R (2004). Habitat differences in allocation of eggs between successive breeding attempts in great tits (*Parus major*).. Ecoscience.

[12] De Laet JF, Dhondt AA (1989). Weight Loss of the Female During the First Brood as a Factor Influencing Second Brood Initiation in Great Tits Parus-Major and Blue Tits Parus-Caeruleus.. Ibis.

[13] Both C (1998). Experimental evidence for density dependence of reproduction in great tits.. Journal of Animal Ecology.

[14] Lindén M (1989). Reproductive trade-off between first and second clutches in the great tit *Parus major*.. Oikos.

[15] Henson MC, Castracane VD, Henson MC, Castracane VD (2003). Leptin and Reproduction.

[16] Fernandez FR, Martini AC, Navarro VM, Castellano JM, Dieguez C (2006). Novel signals for the integration of energy balance and reproduction.. Molecular and Cellular Endocrinology.

[17] Budak E, Fernandez SM, Bellver J, Cervero A, Simon C (2006). Interactions of the hormones leptin, ghrelin, adiponectin, resistin, and PYY3-36 with the reproductive system.. Fertility and Sterility.

[18] Barb CR, Barrett JB, Kraeling RR (2004). Role of leptin in modulating the hypothalamic-pituitary axis and luteinizing hormone secretion in the prepuberal gilt.. Domestic Animal Endocrinology.

[19] Perrins C (1979). British tits.

[20] Smith HG, Källander H, Nilsson J-Å (1987). Effect of experimentally altered brood size on frequency and timing of second broods in the great tit.. Auk.

[21] Tinbergen JM (1987). Costs of reproduction in the great tit: intraseasonal costs associated with brood size.. Ardea.

[22] La Cava A, Alviggi C, Matarese G (2004). Unraveling the multiple roles of leptin in inflammation and autoimmunity.. Journal of Molecular Medicine.

[23] Williams TD (2005). Mechanisms underlying the costs of egg production.. Bioscience.

[24] Tremblay I, Thomas DW, Lambrechts MM, Blondel J, Perret P (2003). Variation in Blue Tit breeding performance across gradients in habitat richness.. Ecology.

[25] Stenseth NC, Mysterud A (2002). Climate, changing phenology, and other life history traits: Nonlinearity and match–mismatch to the environment.. Proceedings of the National Academy of Sciences of the United States of America.

[26] Both C, Bouwhuis S, Lessells CM, Visser ME (2006). Climate change and population declines in a long-distance migratory bird.. Nature.

[27] Visser ME, Both C (2005). Review. Shifts in phenology due to global climate change: the need for a yardstick.. Proceedings of the Royal Society B: Biological Sciences.

[28] Jonzen N, Linden A, Ergon T, Knudsen E, Vik JO (2006). Rapid Advance of Spring Arrival Dates in Long-Distance Migratory Birds.. Science.

